# Knowledge, Behaviours, and Preferred Information Sources Relating to COVID-19 Mitigation Strategies Among Ethnically Diverse Australians

**DOI:** 10.1177/10105395251371252

**Published:** 2025-09-12

**Authors:** Danielle Hutchinson, Aye Moa, Helen Skouteris, Darshini Ayton, Essa Tawfiq, Holly Seale, C. Raina MacIntyre

**Affiliations:** 1 Biosecurity Program, The Kirby Institute, Faculty of Medicine and Health, University of New South Wales, Sydney, NSW, Australia; 2 Health and Social Care Unit, School of Public Health and Preventive Medicine, Monash University, Clayton, VIC, Australia; 3 School of Population Health, University of New South Wales, Sydney, NSW, Australia

## Introduction

The COVID-19 pandemic has had a disproportionately negative impact on migrants and ethnic minority populations globally, with higher standardized death rates and risk of severe disease.^1,2^ Disparities in COVID-19 outcomes for some people from culturally and linguistically diverse (CaLD) backgrounds may be partly due to differences in awareness and uptake of recommended protective behaviors, as well as government communication strategies.^1,2^ The source of health information guiding uptake of mitigation strategies may also influence behaviors. Healey et al^3^ found that low English proficiency may lead community members to rely on English-speaking family members or friends to access and understand COVID-19 information. Watching foreign news and connecting through social media to overseas news sources has been used by CaLD community members to self-educate about COVID-19.^2,3^

This study aimed to estimate differences in knowledge and behaviors about COVID-19 preventive measures between CaLD and non-CaLD groups in Australia, and to assess pandemic information preferences in CaLD and non-CaLD groups in Australia.

## Methods

### Study Design

The “BREATHE” study data set is a result of an anonymous, online, cross-sectional survey of people aged 18 years or over (n = 2867) in Australia conducted in January 2023. A survey link was randomly distributed by the market research company, Dynata, to a geographically targeted national sample of their panel members meeting eligibility criteria.^4^ Data were collected on sociodemographic characteristics, risk factors for COVID-19, knowledge and behaviors regarding infection risk mitigation strategies, and COVID-19 vaccination and infection history.^5^ Responses to questions pertaining to knowledge and behaviors toward COVID-19 mitigation strategies, and preferred COVID-19 information sources was used for this study. The study was approved by the UNSW Human Research Ethics Committee (approval number HC220737).

### Study Participants

We categorized respondents with reference to Australian Bureau of Statistics (ABS) Standards for Statistics on Cultural and Language Diversity.^6^ Respondents were included in the CaLD group if the reported country of birth was a non-main English-speaking country, or if the respondent reported speaking a language other than English at home.

### Statistical Analysis

All data were cleaned prior to analysis, and data analyses were completed using the R Studio software package.^7^ The impact of cultural and language background of respondents on COVID-19 preventive knowledge and behaviors was explored (Table 1). Respondents were instructed to answer “agree” or “disagree” to statements based on their knowledge and uptake of preventive measures SARS-CoV-2 transmission. The scoring rubric is available in Supplemental Table S1.

**Table 1.** Domain Analysis—Comparison Between CaLD and Non-CaLD Groups.

Variable	CaLD (n = 418)	Non-CaLD (n = 2292)	*P* value
**Prevention knowledge**	**N (%)**	**N (%)**	.860
Poor	23 (5.5)	142 (6.2)	
Fair	82 (19.6)	443 (19.3)	
Good	313 (74.9)	1707 (74.5)	
**Prevention practice**	**N (%)**	**N (%)**	**.006**
Weak	155 (37.1)	980 (42.8)	
Fair	162 (38.8)	903 (39.4)	
Strong	101 (24.2)	409 (17.8)	

Bold values indicate statistically significant differences between CaLD and Non-CaLD groups based on Chi-square test (*p* < 0.05).

Respondents were asked to respond to the question “In general, where do you get your COVID-19 news and information from?,” with multiple responses permitted. The data were analyzed using a chi-squared test, comparing observed and expected frequencies of information source preferences between the two groups (Supplemental Table S2).

## Results

The analysis was restricted to the respondents categorized as “CaLD” (n = 418) or “non-CaLD” (n = 2292), constituting the study population (n = 2710) (Figure S1). The distribution of respondents’ prevention knowledge and prevention practice across CaLD and non-CaLD groups was calculated (Table 1). There was no observed difference between groups in the prevention knowledge domain (*P* = 0.86), however there was a significant difference in prevention practices between the groups, with the CaLD group more likely to have a strong commitment to preventive practices (24.2% vs. 17.8%, χ² test, *P* = .006).

**Figure 1.**
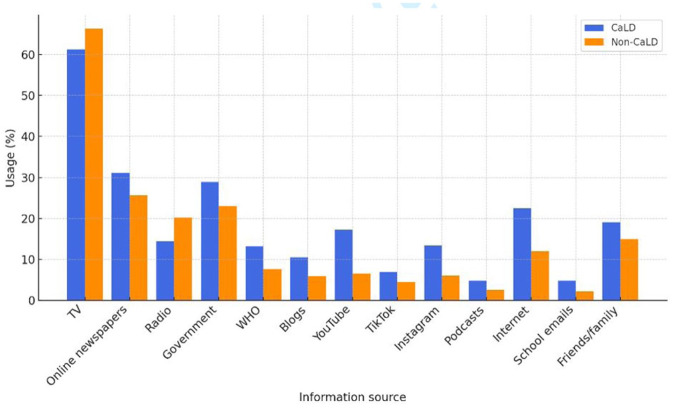
Sources used to access COVID-19 information by CaLD group (more than one response was allowed).

Non-CaLD respondents were more likely to access COVID-19 information from mainstream media and traditional sources, while CaLD respondents were more likely to access COVID-19 information from social media or alternative news sources (Figure 1). CaLD respondents were more likely to use YouTube (17.2% vs 6.5%, *P* < .001), TikTok (6.9% vs 4.4%, *P* = .032), Instagram (13.4% vs 6.0%, *P* < .001), and the internet (22.5% vs 12.0%, *P* < .001) for COVID-19 information (Supplemental Table S2). Those in the CaLD group were also more likely to access COVID-19 information from school (4.8% vs 2.2%, *P* = .004) and family and friends (19.1% vs 14.9%, *P* = .035) (Supplemental Table S2).

## Discussion

We found significant differences in prevention practices and information seeking practices between members of CaLD and non-CaLD groups, with the CaLD group more likely to have a strong commitment to preventive practices. This aligns with prior research in Australia which indicated that people from ethnic minority backgrounds are more likely to engage in health protective behaviors, including mask wearing.^8^

CaLD respondents were more likely to access COVID-19 information from online newspapers, the internet and blogs compared with non-CaLD respondents, and were more likely to use YouTube, TikTok, and Instagram for COVID-19 information. This reliance on online information may be due to unavailability or inadequacy of sources of COVID-19 information in languages other than English, which was outlined in Australian studies early in the pandemic and continued through the vaccine rollout.^2,9^ Seale et al^2^ reported that social media platforms were popular within CaLD communities as a means to access and share COVID-19 information. This may lead to increased susceptibility to misinformation.^10^

Many people from CaLD communities access news and health information from their country of origin, which may lead to missing important local-level information regarding public health strategies.^2^ In addition, CaLD respondents are more likely to seek COVID-19 information from schools (4.8% vs 2.2%) and rely on family and friends (19.1% vs 14.9%). Therefore, it is important that informal sources of information are targeted in public health campaigns aimed at improving knowledge and uptake of preventative behaviors for epidemic control.

There are several limitations in our study. Non-response rates could not be calculated in this study, as Dynata does not provide this data.^5^ Dynata’s open cohort recruitment method may not be representative of the Australian community. The survey was only available in English, which precludes Australians with low English proficiency from providing their information and experiences. However, this large nationwide survey provides a unique perspective to the differences and similarities in the knowledge and uptake of COVID-19 mitigation strategies in CaLD and non-CaLD groups in Australia.

## Conclusion

We found that there was a stronger commitment to preventive practices reported by respondents in the CaLD group, and a greater reliance on online sources of COVID-19 information. Our findings can inform preventive health strategies, including prioritizing preferred information sources, to ensure better outcomes for CaLD populations in health emergencies.

## Supplemental Material

sj-docx-1-aph-10.1177_10105395251371252 – Supplemental material for Knowledge, Behaviours, and Preferred Information Sources Relating to COVID-19 Mitigation Strategies Among Ethnically Diverse Australians Supplemental material, sj-docx-1-aph-10.1177_10105395251371252 for Knowledge, Behaviours, and Preferred Information Sources Relating to COVID-19 Mitigation Strategies Among Ethnically Diverse Australians by Danielle Hutchinson, Aye Moa, Helen Skouteris, Darshini Ayton, Essa Tawfiq, Holly Seale and C. Raina MacIntyre in Asia Pacific Journal of Public Health

## References

[1] Khanijahani A Iezadi S Gholipour K Azami-Aghdash S Naghibi D. A systematic review of racial/ethnic and socioeconomic disparities in COVID-19. Int J Equity Health. 2021;20(1):248. doi:10.1186/s12939-021-01582-4 34819081 PMC8611382

[2] Seale H Harris-Roxas B Heywood AE , et al. “It’s no use saying it in English”: a qualitative study exploring community leaders’ perceptions of the challenges and opportunities with translating and interpreting COVID-19 related public health messaging to reach ethnic minorities in Australia. PLoS ONE. 2024;19(2):e0284000. doi:10.1371/journal.pone.0284000 PMC10903877 38422070

[3] Healey SJR Ghafournia N Massey PD , et al. Factors contributing to the sharing of COVID-19 health information amongst refugee communities in a regional area of Australia: a qualitative study. BMC Public Health. 2022;22(1):1434. doi:10.1186/s12889-022-13850-1 35897090 PMC9331021

[4] Dynata: world’s largest first party data platform. Accessed January 14, 2024. https://www.dynata.com/

[5] Soh SE Ayton D Bevins A Skouteris H Trent M MacIntyre R. Attitudes and behaviours regarding COVID-19 mitigation strategies in Australians with an underlying health condition: a cross-sectional study. Health Expect. 2024;27(5):e70025. doi:10.1111/hex.70025 PMC11391943 39264801

[6] ABS. Standards for Statistics on Cultural and Language Diversity. Australian Bureau of Statistics. Updated February 22, 2022. Accessed March 8, 2024. https://www.abs.gov.au/statistics/standards/standards-statistics-cultural-and-language-diversity/latest-release

[7] Rstudio. RStudio: Integrated Development for R. PBC; 2020. Accessed September 5, 2025. http://www.rstudio.com/

[8] Faasse K Newby J. Public perceptions of COVID-19 in Australia: perceived risk, knowledge, health-protective behaviors, and vaccine intentions. Front Psychol. 2020;11:551004. doi:10.3389/fpsyg.2020.551004 33117223 PMC7561403

[9] McCaffery KJ Dodd RH Cvejic E , et al. Health literacy and disparities in COVID-19-related knowledge, attitudes, beliefs and behaviours in Australia. Public Health Res Pract. 2020;30(4):30342012. doi:10.17061/phrp30342012 33294907

[10] Islam MS Sarkar T Khan SH , et al. COVID-19-related infodemic and its impact on public health: a global social media analysis. Am J Trop Med Hyg. 2020;103(4):1621-1629. doi:10.4269/ajtmh.20-0812 32783794 PMC7543839

