# Supplementary material for: Knowledge, Behaviours, and Preferred Information Sources Relating to COVID-19 Mitigation Strategies Among Ethnically Diverse Australians
Source: Asia Pac J Public Health. 2025 Sep 12;37(6-7):611–4. doi: 10.1177/10105395251371252 (PMC12484627; doi:10.1177/10105395251371252)
Supplement: sj-docx-1-aph-10.1177_10105395251371252 – Supplemental material for Knowledge, Behaviours, and Preferred Information Sources Relating to COVID-19 Mitigation Strategies Among Ethnically Diverse Australians [file sj-docx-1-aph-10.1177_10105395251371252.docx]

**Supplementary Files**


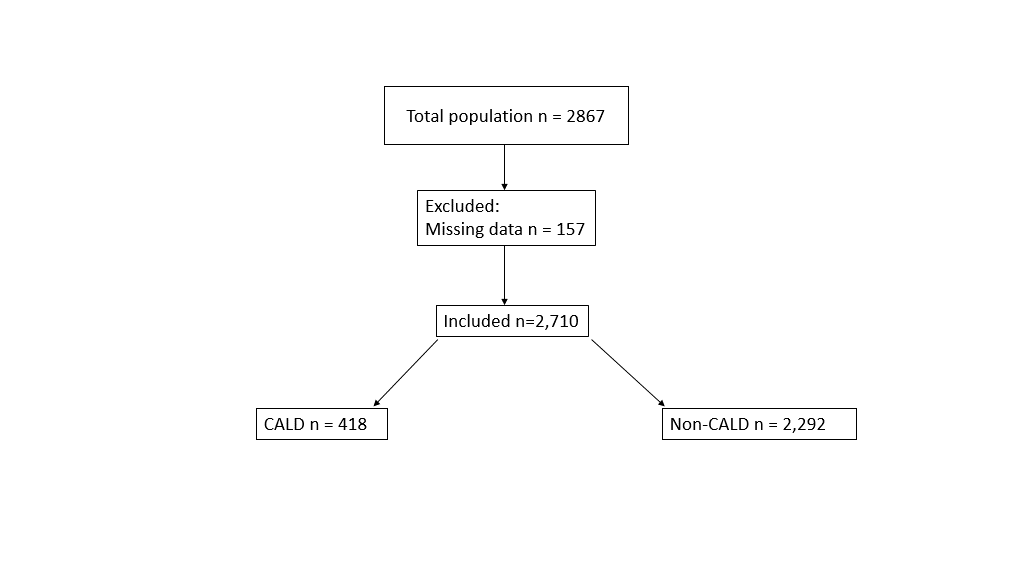


**Supplementary Figure S1. Flow Chart of the Study Population**

**Table S1**

*Scoring rubric for Domain analysis*

| **Domain** | **Score** |
| --- | --- |
| Prevention knowledge (“agree” or “disagree” to eight statements based on their knowledge of preventive measures SARS-CoV-2 transmission) | Poor (0-2 questions correct)  Fair (3-5 answers correct)  Good (6-8 correct) |
| Prevention practice (“agree” or “disagree” to nine statements relating to uptake of preventive measures SARS-CoV-2 transmission) | Weak (0-3 questions agree)  Moderate (4-6 agree)  Strong (7-9 agree) |

**Table S2**

*Sources used to access COVID-19 information by CaLD group*

| **Information source** | CALD (n=418) (%) | Non-CALD (n=2,292) (%) | p-value |
| --- | --- | --- | --- |
| 1.Mainstream/Traditional news source |  |  |  |
| TV news | 256 (61.2) | 1519 (66.3) | 0.053 |
| Online newspapers news | 130 (31.1) | 587 (25.6) | 0.023 |
| Hardcopy newspapers news | 40 (9.6) | 247 (10.8) | 0.515 |
| Radio news | 60 (14.4) | 464 (20.2) | 0.006 |
| Government news | 121 (28.9) | 527 (23.0) | 0.010 |
| WHO news | 55 (13.2) | 174 (7.6) | <0.001 |
| State CMO news | 30 (7.2) | 159 (6.9) | 0.942 |
| State premier news | 24 (5.7) | 138 (6.0) | 0.913 |
| Federal minister news | 22 (5.3) | 89 (3.9) | 0.240 |
| Doctor news | 43 (10.3) | 229 (10.0) | 0.923 |
| 2. Alternative news source |  |  |  |
| Blogs news | 44 (10.5) | 136 (5.9) | 0.001 |
| Twitter news | 14 (3.3) | 91 (4.0) | 0.640 |
| YouTube news | 72 (17.2) | 150 (6.5) | <0.001 |
| Facebook news | 111 (26.6) | 532 (23.2) | 0.157 |
| TikTok news | 29 (6.9) | 100 (4.4) | 0.032 |
| Instagram news | 56 (13.4) | 138 (6.0) | <0.001 |
| Podcasts news | 20 (4.8) | 60 (2.6) | 0.024 |
| Internet news | 94 (22.5) | 276 (12.0) | <0.001 |
| Employer emails news | 40 (9.6) | 156 (6.8) | 0.057 |
| School emails news | 20 (4.8) | 51 (2.2) | 0.004 |
| Friends/family news | 80 (19.1) | 342 (14.9) | 0.035 |

* More than one response was allowed
